# Lighten Up! Postural Instructions Affect Static and Dynamic Balance in Healthy Older Adults

**DOI:** 10.1093/geroni/igz056

**Published:** 2020-03-24

**Authors:** Rajal G Cohen, Jason L Baer, Ramyaa Ravichandra, Daniel Kral, Craig McGowan, Timothy W Cacciatore

**Affiliations:** 1 Department of Psychology and Communication, University of Idaho, Moscow, Idaho; 2 Department of Biological Sciences, University of Idaho, Moscow, Idaho; 3 WWAMI Medical Education Program, University of Idaho, Moscow, Idaho; 4 Sobell Department of Motor Neuroscience, Institute of Neurology, University College London, London, UK

**Keywords:** Aging, Alexander technique, Dance, Electromyography, Embodied mindfulness, Exercise, Feldenkrais, Kinematics, Mobility, Pilates, Posture, Rehabilitation, Tai chi, Yoga

## Abstract

**Background and Objectives:**

Increased fall risk in older adults is associated with declining balance. Previous work showed that brief postural instructions can affect balance control in older adults with Parkinson’s disease. Here, we assessed the effects of brief instructions on static and dynamic balance in healthy older adults.

**Research Design and Methods:**

Nineteen participants practiced three sets of instructions, then attempted to implement each instructional set during: (1) quiet standing on foam for 30 s with eyes open; (2) a 3-s foot lift. “Light” instructions relied on principles of reducing excess tension while encouraging length. “Effortful” instructions relied on popular concepts of effortful posture correction. “Relax” instructions encouraged minimization of effort. We measured kinematics and muscle activity.

**Results:**

During quiet stance, Effortful instructions increased mediolateral jerk and path length. In the foot lift task, Light instructions led to the longest foot-in-air duration and the smallest anteroposterior variability of the center of mass, Relax instructions led to the farthest forward head position, and Effortful instructions led to the highest activity in torso muscles.

**Discussion and Implications:**

Thinking of upright posture as effortless may reduce excessive co-contractions and improve static and dynamic balance, while thinking of upright posture as inherently effortful may make balance worse. This may partly account for the benefits of embodied mindfulness practices such as tai chi and Alexander technique for balance in older adults. Pending larger-scale replication, this discovery may enable physiotherapists and teachers of dance, exercise, and martial arts to improve balance and reduce fall risk in their older students and clients simply by modifying how they talk about posture.

**Translational Significance:**

Thinking of upright posture as effortless reduced muscle activation and improved balance, while thinking of upright posture as effortful made balance worse. This may partly explain the benefits of embodied mindfulness practices such as tai chi and Alexander technique for balance in older adults. Pending larger-scale replication, these findings suggest that physiotherapists and teachers of dance, exercise, and martial arts may improve balance and reduce fall risk in their older students and clients by using this information to inform how they talk about posture.

## Background and Objectives

Older adults tend to be prone to falls, which can have large physical and financial costs (1–3). Risk of falling is associated with declines in static and dynamic balance, which are also common in older adults (4–6). Therefore, the development of practical ways to improve balance is vital to reduce falls, injury, and long-term disability in older adults. Exercise-based falls-prevention interventions are promising (7), but results are uneven (8). Research is needed on characteristics of interventions that may contribute to successful outcomes.

Some successful fall-risk interventions (e.g., tai chi and dance) are notable not only for their physical vigor but also for their requirement that participants attend to the quality of their *postural state* (9–12). Postural state includes postural alignment (the arrangement of body parts in relation to one another and to gravity) and postural tone (the distribution of persistent muscular activation throughout the body axis). Postural state affects static and dynamic balance (control of center of mass with respect to base of support, during quiet stance and during movement) (13–17). General evidence for the importance and modifiability of postural state (especially postural tone) comes from studies demonstrating that people with chronic neck pain have a different distribution of postural tone in their spinal musculature than people without pain, and that this pattern can be altered with guided practice and attention (18,19). Thus, quality of attention to postural state may be an important mechanism by which effective interventions influence balance.

A previous study found that attention to postural state affected postural alignment and postural tone, as well as static and dynamic balance in participants with Parkinson’s disease (20). That study used three sets of instructions. The Light (or “lighten up”) instructions were based on the principles of Alexander technique, an approach to posture known to affect postural tone and movement coordination (19,21–25). The idea is to reduce excessive muscle activation while maintaining spinal length and a sense of connection throughout the body. The Effortful (or “pull up”) instructions were based on prevalent public conceptions of “good posture” as something requiring effort. For instance, popular advice available on Internet websites published by mainstream medical establishments emphasizes pulling the head up (26), holding the back straight (27), squeezing the shoulder blades together (28,29), and tightening the abdominal muscles (28,30). The Relax instructions were based on the widespread idea that maintaining upright posture is inherently fatiguing. Results indicated that the Light instructions reduced axial stiffness, decreased postural sway, and improved control during stepping compared to Relax instructions, while the Effortful instructions did not improve any measured aspect of balance.

The previous work could not determine whether the balance improvements seen following Light instructions depended on the deficits in postural regulation and balance associated with Parkinson’s disease, which manifest in exaggerated forward stooping (31), high stiffness (32), and increased postural sway (33). However, normal aging also leads to forward stooping (16,17), high muscle stiffness (34,35), and increased postural sway (36,37), all of which could increase fall risk. Therefore, the present study extends the previous work by asking how different ways of attending to postural state might affect balance in healthy older adults.

Due to differences in laboratory equipment available, not all tasks from the previous study could be exactly replicated. Thus, the present study includes one replicated task (quiet stance with inertial sensors) and one new task (3-s foot lift). The foot-lift task was included because it challenges lateral balance; sideways falls are especially problematic for older adults, as they often lead to hip fractures (38).

In this project, as in the previous study, we investigated three different instructions aimed to affect postural state. The “Light” condition provides a gentle invitation to allow the head to balance lightly on the top of the spine and prevent any downward pulling. The “Effortful” condition exhorts participants to use muscular effort and “core strength” to pull their heads up to their full height and their shoulders back. The “Relax” instructions encourage minimization of effort with no specific intention of postural uprightness. The purpose of the study was to evaluate the effects of specific postural instructions on balance in healthy older adults. Based on previous findings, we predicted that both the Light instructions and the Effortful instructions would improve postural alignment, but that the Effortful instructions would also increase muscle coactivation, thus interfering with static and dynamic balance.

## Research Design and Methods

### Recruitment and Screening

We recruited healthy adults over age 60 by flyer, radio, university email, and social media. To be included, participants had to be able to hear and understand instructions, and to indicate that they could stand independently for 20 min without major discomfort.

### Procedure

**Figure 1.**
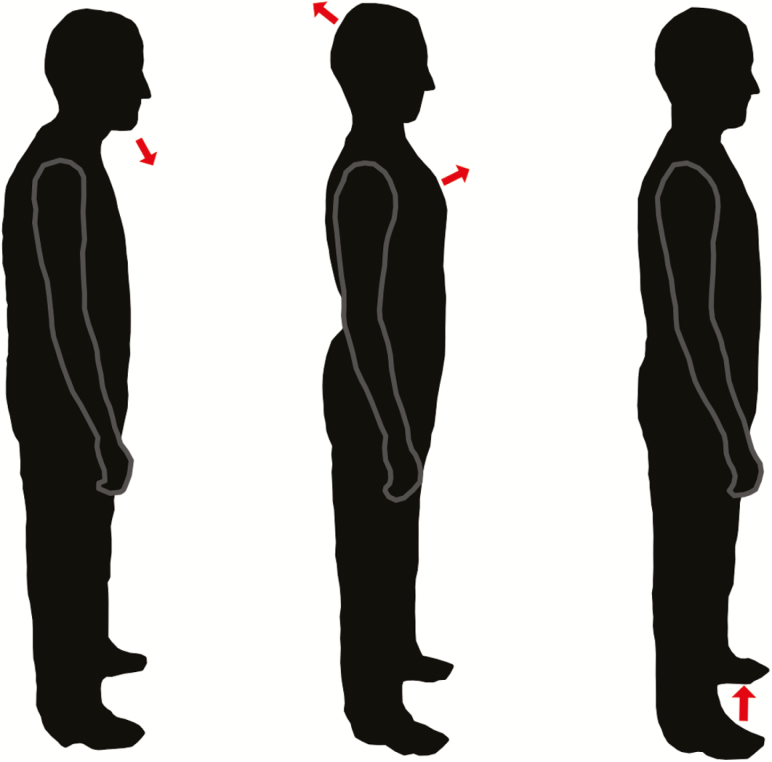
Illustration of the sort of posture participants adopted in response to the three different instructions. Left: Relax. Middle: Effortful. Right: Light.

After signing consent forms approved by University of Idaho’s Internal Review Board, participants were instructed and given several minutes of practice in each of three postural conditions: Light, Effortful, and Relax. To ensure consistency, experimenters (research assistants with no special medical training) read from a standard script (middle column of Table 1). Participants practiced transitioning among the sets of instructions until they felt confident that they could clearly distinguish all three conditions (see Figure 1). They then performed two tasks in each postural condition: (1) stand quietly for 30 s, with a three-dimensional inertial sensor attached to the lumbar region to quantify postural sway; (2) lift one foot off the floor and hold it for 3 s, wearing reflective markers to record kinematics and electromyography (EMG) to quantify muscle activity on the stance side of the body.

**Table 1.** Verbatim Instructions for Each Condition

Postural instructions	Full version	Short version
**Relax**	Stand as you would if you were feeling tired and lazy; like it’s the end of a day, and nobody is watching, and you do not really care about your posture. Let your head and chest feel heavy and let everything settle a bit downward.	Stand relaxed and heavy and let everything settle down.
**Effortful**	Use muscular effort to pull yourself up to your greatest height. Pull your head up, lift your chest, and tighten all the core muscles in your torso. You can think of holding a military posture, which *looks* really strong. Really work at it!	Pull yourself up to your greatest height, using muscular effort.
**Light**	Have the idea that you WANT to go up, but you are not going to do it with muscular effort. Instead, let the ground send you up through your bones, and let your head float up on top of your spine. (Remember where we touched you behind the ears when we were setting up the camera system? The top of your spine is right between those points.) Notice that at the same time as you are going up, you can also expand into width.	Allow your bones to send you up; let your head float on top of your neck.

The order of conditions was fully counterbalanced. Participants completed three consecutive trials all conditions in each condition before changing conditions, and they completed the first task before beginning the second task. At the start of each trial, the experimenter provided a very brief review of the postural instructions for that condition (last column of Table 1). A research assistant stood close to the participant to prevent falls if necessary. While performing the tasks, participants were instructed to keep their eyes open and to look straight ahead at a poster on the wall.

After completing both tasks, participants filled out a brief subjective assessment about their experiences of the different postural conditions, using a 0–4 scale to indicate the familiarity of each postural condition, the effect of each condition on their sense of stability, and the degree of mental and physical effort required in each condition.

### Tasks, Measures, and Analyses

Participants stood facing a wall with a decorative poster on it, 2.5 m away. They were instructed to look forward during task performance. No visual fixation was provided (39).

To assess static balance, we examined sway during quiet stance. Participants stood on a springy foam mat (Airex, Switzerland) with their arms crossed in front of their chests for 30 s; an inertial sensor (APDM, Portland, OR) was attached by a waist strap to their lumbar region. Outcome measures reported by the inertial sensors were root mean square amplitude (m.s^−2^), frequency (Hz), path length (m^2^.s^−2^), and mean squared jerk (m^2^.s^−5^) in the mediolateral (ML) and anteroposterior (AP) axes (33). Examining the data, we observed that one participant had extremely high sway (over 4 *SD* above the mean of the group in all measures). That participant’s data were removed and replaced with the next highest value for each outcome measure. (This process, known as Winsorization, is more conservative than simply trimming outliers (40).)

**Figure 2.**
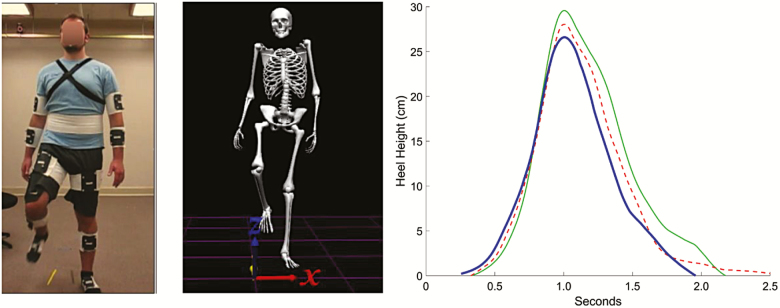
Foot lift task. Left image shows peak of foot lift task (frontal view) modeled by a young research assistant, wearing the reflective marker clusters. Middle image shows the peak foot lift based on motion capture data. Right image shows mean heel height across participants during foot lift trials in three conditions. Solid green line = Light. Red dashed line = Effortful. Heavy blue line = Relax. The red line does not return to zero because in the Effortful condition, some participants did not return their heels all the way to the ground.

To assess dynamic balance, we examined kinematics and muscle activity during an in-place foot lift. Participants were instructed to raise the left foot off the floor, hold for 3 s, and put it back down. Trials began 1 s before the experimenter said, “Go ahead,” and lasted for 6 s. We used eight motion capture cameras with Nexus software (Vicon, Oxford, UK) to track clusters of reflective markers grouped into 14 body segments (Figure 2). Data were streamed at 100 frames/s to The MotionMonitor software (Innovative Sports Training, Chicago, IL). The MotionMonitor used these markers and segments to generate a model of each participant’s body and the location of their center of mass (CoM) over the course of each trial. We then used MATLAB (MathWorks, Natick, MA) to compute the duration the toe was in the air (ms), the peak height of the heel during the foot lift (cm), the maximum horizontal distance of the head (mastoid process) forward from the base of the neck (seventh cervical vertebra) and the range of that distance (cm), the minimum vertical distance of the head above the base of the neck and the range of that distance (cm), the standard deviation of the position of the CoM in ML and AP axes (cm), the mean square jerk of the CoM in ML and AP axes (m^2^.s^−5^), and the total range (deg) of the pelvis twist around the vertical axis and pelvis tilt in the frontal plane.

For the dynamic task, Bagnoli Ag/AgCl surface electrode units (Delsys, Natick, MA) were attached over five muscles on the right (stance) side of the body: tensor fasciae latae, gluteus medius, external oblique, and longissimus and iliocostalis at the level of the third lumbar vertebra. Prior to electrode placement, skin was prepped by shaving, lightly abrading, and cleansing with rubbing alcohol. Electromyographic data were recorded at 1,000 samples/s, filtered, and rectified. We computed peak activity and integrated EMG activity for each muscle during each trial to assess phasic and tonic changes in muscle activation. Because all comparisons were within-participants and within-session, the data were inherently normalized.

For each outcome measure, we selected median values for each participant from each condition and conducted single-factor analysis of variance (ANOVA). For variables in which a difference was detected by ANOVA (*p* < .05), Tukey-corrected post hoc comparisons were performed (41).

## Results

### Participants

We tested 19 healthy adults (7 men and 12 women) between 60 and 80 years of age (mean 69 years). On average, participants reported that they spent 5.7 hr per day sitting and 40 min per day exercising (with respective *SD*s of 2.8 hr and 26 min) and had 4.2 years postsecondary education (*SD* 2.4 years). Mean (and *SD*) height and weight were 167 (8.6) cm and 74.3 (16.1) kg, respectively.

### Quiet Stance

Table 2 shows the effects of the different instructions on postural sway during quiet stance. Postural instructions had no significant effect on amplitude or frequency in either axis. However, ML path length was higher in the Effortful condition than in Light or Relax conditions, and AP path length showed a similar, though nonsignificant, pattern. ML jerk was 33% higher in the Effortful condition than in the other two conditions.

**Table 2.** Postural Sway Results

	Mean (*SD*)			*F* (*p*)	Tukey post hoc		
	Light	Effort	Relax		L vs E	E vs R	L vs R
RMS—AP	.13 (.04)	.14 (.04)	.12 (.04)	2.4 (.11)	-	-	-
RMS—ML	.059 (.02)	.062 (.02)	.055 (.02)	2.3 (.12)	-	-	-
Freq—AP	.57 (.10)	.55 (.12)	.59 (.23)	0.7 (.52)	-	-	-
Freq—ML	.82 (.25)	.86 (.19)	.82 (.22)	0.5 (.63)	-	-	-
Path—AP	8.33 (3.74)	9.59 (4.42)	8.77 (5.58)	2.9 (.07)	-	-	-
**Path–ML**	**6.14 (2.12)**	**7.00 (2.77)**	**5.47 (1.72)**	**10.3 (.0003)**	**NS**	*	*
Jerk—AP	.025 (.020)	.031 (.031)	.029 (.042)	1.2 (.32)	-	-	-
**Jerk–ML**	**.012 0 (.008)**	**.016 (.012)**	**.012 (.012)**	**3.9 (.03)**	**NS**	*	**NS**

*Note*: Reported values are across-participant means of each participant’s median value in each condition. RMS = root mean square (a measure of amplitude, m.s^-2^). Path = path length (a measure combining amplitude and velocity, m^2^.s^−2^). Freq = median frequency (Hz). Jerk = mean squared jerk (inverse of smoothness, m^2^.s^−5^). AP = anteroposterior. ML = mediolateral. NS = not significant. Degrees of freedom = (2,37).

*p < .05. NS = *p* > .05.

### Foot-Lift Task

Kinematics are shown in Table 3. The Light condition led to the longest foot-in-air duration, while the Effortful condition led to the shortest duration. Importantly, there was no difference in the peak foot height across conditions, indicating that the longer duration of the foot lift in the Light condition was not due to a more conservative (less vigorous) strategy (Figure 2).

**Table 3.** Kinematics from Foot-Lift Task

	Means (*SD*)				Tukey post hoc		
	Light	Effortful	Relax	*F* (*p*)	L vs E	E vs R	L vs R
**Foot in Air (ms)**	**2528 (728)**	**2207 (558)**	**2333 (592)**	**4.4 (.019)**	*	**NS**	**NS**
Peak Foot Height (cm)	30.0 (10.2)	28.6 (11.9)	27.0 (11.9)	1.0 (.37)	-	-	-
**Head forward max (cm)**	**1.3 (2.3)**	**1.0 (2.2)**	**1.9 (2.5)**	**5.2 (.01)**	**NS**	**NS**	**NS**
Head forward range (cm)	1.00 (.43)	0.99 (.39)	1.01 (.39)	.025 (.98)	-	-	-
Head vertical min (cm)	12.1 (2.0)	11.9 (1.9)	12.0 (1.9)	0.7 (.49)	-	-	-
Head vertical range (cm)	0.52 (0.33)	0.58 (.23)	.56 (.26)	0.4 (.66)	-	-	-
CoM Jerk—ML (m^2^.s^–5^)	.278 (.245)	.350 (.366)	.368 (.496)	0.6 (.57)	-	-	-
CoM Jerk—AP (m^2^.s^–5^)	.073 (.093)	.154 (.438)	.158 (.414)	0.7 (.51)	-	-	-
CoM SD—ML (cm)	4.89 (1.18)	4.97 (1.00)	5.08 (1.41)	0.6 (.58)	-	-	-
**CoM SD—AP (cm)**	**0.69 (0.33)**	**0.94 (0.54)**	**0.95 (0.54)**	**5.2 (.01)**	*	**NS**	**NS**
Pelvis twist range (deg)	6.89 (2.76)	7.52 (2.77)	7.88 (2.14)	2.6 (.09)	-	-	**-**
Pelvis tilt range (deg)	5.62 (2.08)	5.70 (1.56)	5.77 (1.98)	0.1 (.89)	-	-	-

*Note:* Reported values are across-participant means of each participant’s median value in each condition. CoM = center of mass. Jerk = mean squared jerk. ML = mediolateral. AP = anteroposterior. NS = not significant. Degrees of freedom = (2,36), except for Foot in Air, which has (2,34) degrees of freedom due to a technical glitch in one participant’s data.

*p < .05. NS = *p* > .05.

**Figure 3.**
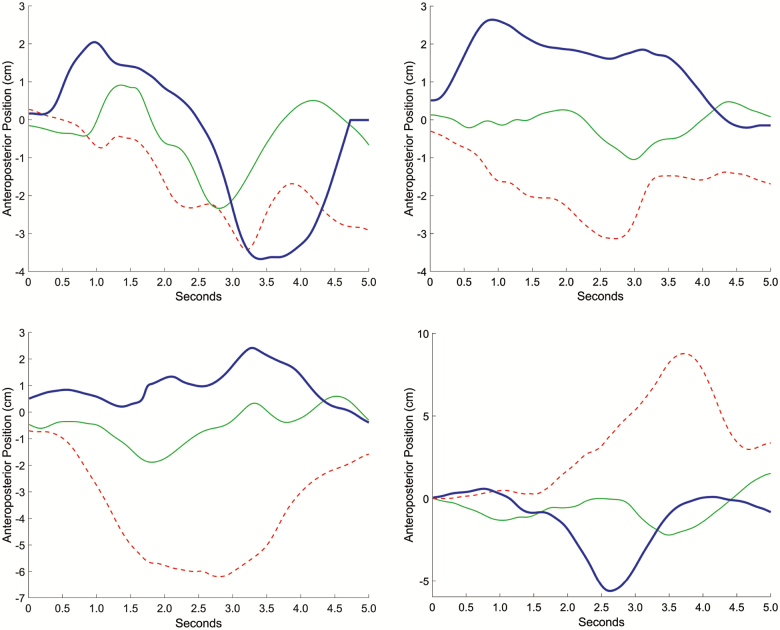
Anteroposterior center of mass. Representative traces of center of mass position in anteroposterior axis with respect to the ankle during individual foot lift trials. Data from four different participants are shown in three conditions. Red dashed line = Effortful. Solid green line = Light. Heavy blue line = Relax.

The Light condition led to the smallest AP variability of the CoM (27% lower than the Effortful condition). Several representative trials are shown in Figure 3. The absence of a consistent pattern in the AP movement of the CoM suggests that smaller variability in this measure may indicate better balance control. The Light condition also seemed to lead to lower jerk (in both axes) and less pelvis twist than the other two conditions, but none of these differences reached significance.

The Relax condition appeared to lead to the most forward head relative to the neck, although this difference was not significant in post hoc comparisons. There was no difference in the relative range of head position, suggesting that the instructions altered the baseline posture and this altered posture was maintained similarly during movement in all conditions.

Muscle activity is shown in Table 4. During the foot lift task, torso muscle activity on the stance side of the body was higher in the Effortful condition than in the other two conditions. Specifically, the Effortful instructions led to the highest total activity (integrated EMG) for the iliocostalis muscle at the L3 level, and to the highest peak activity in the external oblique muscle. Note that this greater muscle activity in the Effortful condition did not lead to a higher foot lift or longer duration, as the Light condition led to the longest duration and the (nonsignificantly) highest foot lift.

**Table 4.** Muscle activity during foot lift task

		Mean (*SD*)				Tukey post hoc		
		Light	Effortful	Relax	*F* (*p*)	L vs E	E vs R	L vs R
iEMG	TFL	0.033 (0.026)	0.026 (0.020)	0.024 (0.017)	2.3 (.12)	-	-	-
	Gmed	0.017 (0.017)	0.021 (0.023)	0.022 (0.016)	0.9 (.73)	-	-	-
	ExtObl	0.020 (0.015)	0.031 (0.022)	0.027 (0.028)	1.7 (.19)	-	-	-
	**LongL3**	**0.021 (0.015)**	**0.033 (0.025)**	**0.018 (0.014)**	**3.7 (.03)**	**NS**	**NS**	**NS**
	**IlioL3**	**0.016 (0.020)**	**0.029 (0.027)**	**0.016 (0.013)**	**6.5 (.004)**	*	*	**NS**
Peak	TFL	0.376 (0.353)	0.406 (0.328)	0.380 (0.355)	1.3 (.30)	-	-	-
	Gmed	0.268 (0.188)	0.270 (0.259)	0.251 (0.230)	1.2 (.32)	-	-	-
	**ExtObl**	**0.090 (0.053)**	**0.115 (0.069)**	**0.096 (0.055)**	**1.2 (.002)**	*	**NS**	**NS**
	LongL3	0.128 (0.118)	0.157 (0.152)	0.133 (0.091)	2.3 (.12)	-	-	-
	IlioL3	0.118 (0.089)	0.138 (0.102)	0.122 (0.098)	2.2 (.13)	-	-	-

*Note:* Reported values are across-participant means of each participant’s median value in each condition. iEMG = integrated electromyographic signal (total activation during trial). TFL = tensor fasciae latae. Gmed = gluteus medius. ExtObl = external oblique muscle. LongL3 = longissimus muscle at level of third lumbar vertebra (L3). IlioL3 = iliocostalis at L3 level. NS = not significant. All muscles recorded are on the stance side of the body (arbitrary units). Degrees of freedom = (2,36).

*p < .05. NS = *p* > .05.

### Subjective Assessment

Participant impressions of the instructions are shown in Table 5. Participants rated all three sets of instructions as equally familiar and stable. They rated the Effortful condition as requiring more mental and physical effort than the other two conditions. Two participants did not complete the subjective assessment, due to experimenter error.

**Table 5.** Subjective Assessment

	Mean				Tukey post hoc		
	Light	Effortful	Relax	*F* (*p*)	L vs E	E vs R	L vs R
Familiarity	3.24	3.18	3.24	0.0 (.98)	-	-	-
Stability	3.18	2.88	3.18	0.9 (.42)	-	-	-
**Mental effort**	**2.35**	**3.00**	**1.88**	**7.0 (.003)**	*	*	**NS**
**Physical effort**	**1.59**	**3.00**	**1.76**	**9.1 (.00007)**	*	*	**NS**

*Note*: Ratings are on a 0–4 scale. NS = not significant. Degrees of freedom = (2,32).

*p < .05. NS = *p* > .05.

## Discussion and Implications

### Interpretation of Findings

This study used a counterbalanced repeated-measures design to investigate how balance would be affected by instructing healthy older adults to stand with a Relaxed, Effortful, or Light postural intention. Both the Effortful and the Light instructions led to a more upright postural alignment (as indicated by how forward the head was with respect to the body) compared to the Relax condition. However, the two different ways of thinking about upright posture led to divergent effects on balance.

During quiet standing, Effortful instructions led to the jerkiest ML movement of the CoM with the longest path length. Both high jerk and high path length during quiet stance are associated with poor stability (33,42). Therefore, these results suggest that using voluntary muscular effort to pull oneself up to one’s greatest height interferes with static balance control. Subjective reports and electromyography both indicated that participants exerted the most muscular effort during this condition. The resulting co-contraction may have interfered with participants’ ability to make rapid, minute, automatic adjustments to balance. This explanation is consistent with recent work suggesting that delays in reaction times in older adults may be associated with muscular co-contraction (43).

When attempting to perform a 3-s standing foot lift, Light instructions led to the longest foot-in-air duration (321 ms longer than Effortful instructions) and the least variable AP CoM. Standing longer on one foot with less CoM disturbance indicates better dynamic balance (44,45). Therefore, our results overall suggest that maintaining an easy upward intention improves static and dynamic balance. By reducing excess co-contraction while encouraging antigravity support, the Light instructions may lead to a postural state that facilitates automatic modulation of postural tone, as was observed previously with similar instructions in participants with Parkinson’s disease. This explanation is consistent with results from a neuromechanical model showing that better control of postural tone is associated with better control of movement (22).

### Comparison to Previous Findings

This study extended to healthy older adults the approach used in a previous study of people with Parkinson’s disease. Due to laboratory differences, a 3-s foot-lift task was substituted for step initiation, and muscle activity was assessed instead of axial compliance. Overall, the results were consistent across studies, with Light instructions improving balance and Effortful instructions making it worse. The results are also consistent with the findings of another study in which instructing dancers with back pain to envision holding themselves in a “gentle, lifted way” led to improved trunk dynamics (46).

By converting the present study’s subjective ratings to a 0–10 scale (by multiplying by 2.5), we can compare them directly to the previous results. In the previous study, participants with Parkinson’s disease reported that the Light instructions were far less familiar than the other instructions (rating them around 4 on a 0–10 scale, with the other instructions rated around 8), whereas the healthy older adults in the present study rated all three instructions around 8 on the same scale. This may reflect subtle differences in the delivery of the instructions. In the previous study, a trained Alexander teacher delivered the instructions interactively, without a script, while in the present study, experimenters with no Alexander training read the instructions from a script. This change was implemented to ensure that any effects were due to the instructions themselves, rather than to subtle pedagogical factors. However, the greater level of interaction in the previous study may have helped participants to better grasp the differences between the instructions.

### Strengths and Limitations of the Approach

Because postural state can be changed very quickly (relative to, for instance, muscular strength or reflex speed), we can have participants alternate between the different conditions in the course of a single session. Comparisons between treatments are made within the same participant, which eliminates confounds associated with assigning participants to groups and improves statistical power. This approach also allows us to keep the instructions very consistent. Our findings do not tell us about long-term learning and retention of this sort of postural tone intervention; however, long-term changes in postural tone have been reported from a full course of 20 Alexander technique lessons (which also included hand contact) (25).

The delivery of instructions from a script was implemented to improve experimental control. However, the finding that the participants in the present study were less likely than those in the previous study to recognize the Light instructions as novel suggests that the scripted instructions may not have been as effective at inducing change in postural state as instruction delivered by a trained Alexander teacher.

As noted in the Analysis section, one participant had very large postural sway (more than 4 *SD* above the group mean in all measures). It is possible that this person had an impairment such as vestibular dysfunction or peripheral neuropathy. Future studies might screen for these conditions.

### Theoretical Implications and Future Directions

Age-related increases in stiffness and muscle coactivation have been shown for the elbow (47), knee (48), and ankle (49) joints, indicating a decreased adaptability of postural tone. The present results suggest that this increased coactivation may contribute to the decreased balance typically seen in older adults, perhaps through neuromechanical interference (22). Furthermore, aging is associated with changes in cortical activity during posture and locomotion. Evidence of an age-related increase in multisensory cortical control suggests that voluntary strategies may play an increasing role in postural control with age (50). Therefore, clarifying postural intentions may become increasingly important in older adults.

It is well known that emphasizing attention to a body part directly involved in an action can impair performance (51,52). In our studies, the instructions focused on the torso and neck, but the outcomes assessed changes in control of the center of mass. Furthermore, the instructions were only about postural state, but the outcome measures were about balance, thus decoupling the instructions from the task. The indirectness of the postural instructions used here may avoid the problems of interference caused by direct focus seen in the work of Wulf and associates (51,52), allowing for positive effects to emerge. Another benefit of addressing postural state is that instructions are not task-dependent and thus improvements may generalize across tasks.

Note that head position, per se, cannot explain our results. Head position was not significantly different between the Light and Effortful conditions, where we found some of the largest outcome differences: relative to the Light condition, the Effortful condition led to a 33% increase in ML jerk during quiet stance; and a 28% increase in peak external oblique activity, 81% increase in total iliocostalis activity, 321 ms decrease in foot lift duration, and 36% increase in variability of AP center of mass during the foot-lift task. Our results are instead consistent with the idea that postural tone has global effects via kinematic chains throughout the body (20,53).

### Translational Implications

The Light instructions developed for this study were simplified to resemble instructions a participant could receive in a dance or exercise course. However, the instructions were initially derived from Alexander technique, a systematic method for developing sensitive control that is commonly used by actors, dancers and musicians to improve performance (54–57). Students of Alexander technique learn to notice and prevent postural habits, especially those involving excessive muscular tension, that interfere with efficient movement. Randomized controlled studies have found that a course of 20–24 Alexander lessons reduces back pain in chronic sufferers (58), improves mobility in people living with Parkinson’s disease (59,60), and increases respiratory capacity (61), while 8–12 lessons can reduce postural sway (62) and increase functional reach (63) in older adults. A course of Alexander lessons was also found to improve postural tone in people with chronic back pain, and more extensive training was shown to improve it further (25). Therefore, the present results support the growing consensus that learning to maintain particular intentions with regard to one’s postural state can have widespread benefits.

The success of our instructions at affecting postural state and balance suggests that use of such instructions may be a promising direction for rehabilitation interventions. Instructors of approaches such as tai chi, yoga, dance, Feldenkrais, Pilates, and Alexander technique often direct participants to think about posture and quality of movement in particular ways (9). Those instructions may play a key role in the effectiveness (or lack thereof) of the approaches. Note that the different instructions used in this study had distinct effects on postural alignment and balance. The Effortful instructions, which were based on popular conceptions of good posture, led to the worst overall static and dynamic balance. This is important, as it suggests that effortful cueing of posture during activity-based therapies and trainings may actually have a negative impact on performance and fall risk. Our results are consistent with emerging evidence that effortful “straight” posture is not as helpful as it has been thought to be (64).

### Summary and Conclusions

The results of this study are important for older adults because they directly address a root cause of falling, that is, poor balance. Our approach targets aspects of postural state that are known to decline with aging and that may be fundamentally related to balance control. Older adults often have a forward stooped posture (65), increased muscle coactivation (48), and increased postural sway (6), all of which have been associated with impairments in balance (17). If, as our results suggest, instructions that encourage an effortless upward intention can decrease excessive muscle coactivation and improve balance in older adults, these instructions should be widely integrated into rehabilitation programs. In addition, an intervention that does not require the ongoing setting aside of time for a regular activity could be beneficial for a majority of the aging population, including those for whom an exercise program is not appropriate as well as those who already exercise regularly.
